# Physical activity, exercise and adverse cardiovascular outcomes in individuals with pre-existing cardiovascular disease: a narrative review

**DOI:** 10.1080/14779072.2024.2328644

**Published:** 2024-03-15

**Authors:** Setor K. Kunutsor, Jari A. Laukkanen

**Affiliations:** a Leicester Real World Evidence Unit, Diabetes Research Centre, University of Leicester, Leicester General Hospital, Leicester, UK; b Institute of Public Health and Clinical Nutrition, University of Eastern Finland, Kuopio, Finland; c Institute of Clinical Medicine, Department of Medicine, University of Eastern Finland, Kuopio, Finland; d Wellbeing Services County of Central Finland, Department of Medicine, Jyväskylä, Finland

**Keywords:** Physical activity, exercise, cardiovascular disease, mortality, cohort study, randomized controlled trial

## Abstract

**Introduction:**

The evidence supporting the cardiovascular health benefits of physical activity and/or exercise training is well-established. While the role of physical activity in primary prevention is unequivocal, its significance in secondary prevention (among those with preexisting cardiovascular disease) is less definitive. Though guidelines universally recommend physical activity as part of the secondary preventive strategy, the empirical evidence underpinning these recommendations is not as robust as that for primary prevention.

**Areas covered:**

This review distills the body of available observational and interventional evidence on the relationship between physical activity, exercise, and adverse cardiovascular outcomes among those with preexisting cardiovascular disease. The postulated biologic mechanisms underlying the relationships, areas of prevailing uncertainty, and potential public health implications are also discussed.

**Expert opinion:**

A physical activity level of 500 MET-min/week (equivalent to 150 min of moderate-intensity physical activity or 75 min of vigorous-intensity physical activity or an equivalent combination) may be a minimum requirement for patients with preexisting CVD. However, to reap the maximum benefits of physical activity and also minimize adverse effects, physical activity and/or exercise regimens should be tailored to unique factors such as individual’s baseline physical activity habits, cardiovascular health status and the specific nature of their cardiovascular disease.

## 1. Introduction

Cardiovascular diseases (CVDs), which encompass a range of conditions that affect the heart and blood vessels, remain the leading causes of morbidity and mortality globally [1]. Atherosclerotic CVD (ASCVD), which manifests mostly as coronary heart disease (CHD) and stroke, is the major clinical manifestation of CVD. Other cardiovascular conditions include heart failure (HF), peripheral artery disease, and arrhythmias, which include atrial fibrillation (AF) and ventricular tachycardia. Millions of people are affected by any of these CVDs annually, resulting in a significant strain on healthcare systems and economies worldwide [2,3]. The World Health Organization (WHO) has consistently highlighted CVD as a primary concern due to its widespread prevalence and high association with preventable deaths. As populations age and lifestyles change, the epidemiology of CVD continues to evolve, necessitating a comprehensive approach to understanding and mitigating its impact. Leading environmental, metabolic, and behavioral risks for CVDs include ambient particulate matter, air pollution, household air pollution from solid fuels, lead exposure, low or high temperature, elevated systolic blood pressure, high low-density lipoprotein cholesterol, high body mass index (BMI), high fasting plasma glucose, kidney dysfunction, dietary risks, tobacco smoking, secondhand tobacco smoke, excessive alcohol use, and low levels of physical activity [4]. Given the significant challenges posed by CVDs in the United States (US), the American Heart Association (AHA) in 2010 developed a cardiovascular health metric to improve the cardiovascular health of all Americans, while reducing deaths from CVDs and stroke. The metric termed Life’s Simple 7 comprises health behaviors (smoking, physical activity and diet) and health factors (blood pressure, BMI, blood glucose and blood cholesterol) which need to be positively modified to achieve ideal cardiovascular health [5]. In 2022, Life’s Simple 7 was upgraded to the Life’s Essential 8 to enhance the approach of achieving optimal cardiovascular health; this update included more refinement of the original 7 components as well as the addition of sleep health to reflect its important role and impact on cardiometabolic health [6]. The AHA’s Life’s Essential 8 highlights physical activity’s central role in CVD prevention at an individual and population level; this is because physical inactivity is a major threat to cardiovascular health. In 2021, 0.397 million cardiovascular deaths and 0.686 million deaths overall were estimated as being attributable to inadequate physical activity [4]. Given the low levels of physical activity globally, the WHO has developed a new global action plan to help countries scale up policy actions to promote physical activity; the goal is to reduce physical inactivity by 10% by 2025 and 15% by 2030 [7].

For the purposes of this review, it was essential to define and clarify some relevant terminologies that would feature a lot, and these include physical activity, exercise, physical inactivity, and sedentary behavior. ‘Physical activity’ and ‘exercise’ are terms that are commonly used interchangeably, but they are not necessarily the same. Physical activity is defined as any skeletal muscle movement that requires energy expenditure and includes exercise, leisure time activity as well as usual occupational and/or domestic activity [8]. In contrast, exercise is intentional physical activity that is designed to improve physical fitness and can include aerobic, high-intensity interval, or resistance training [9].

Physical inactivity is not the exact inverse of physical activity and is typically defined as not meeting specified physical activity guideline recommendations [8,9]. Furthermore, sedentary behavior, also commonly used interchangeably as physical inactivity, is defined as any waking activity characterized by low metabolic energy expenditure (≤1.5 metabolic equivalents, METs) while in a sitting, reclining or lying posture [10,11]. Components of physical activity include the frequency, duration, and intensity, which comprise the total volume [12–14]. Frequency involves the number of physical activity/exercise sessions in a specific time period (e.g. per week), duration is the time spent for each physical activity/exercise session, intensity refers to exertion during physical activity/exercise and is a reflection of energy expenditure, and volume refers to the product of frequency, duration, and intensity [15]. Physical activity can be classified by the level of intensity: light (or low), moderate, and vigorous (Table 1). The unit of METs is a measure for absolute intensity and reflects energy expenditure during rest (which approximates to 3.5 ml of oxygen uptake per kilogram of body weight per minute for the average adult) [10]. Light-intensity activities require 1.1–2.9 METs and include activities such as walking at a pace of less than 2.0 miles per hour or 1 mile in 30 min; moderate-intensity activities are those that require 3–5.9 METs and include walking 3.5 miles per hour or at a pace of 1 mile in 17 min; and vigorous-intensity activities require ≥ 6 METs and include running 1 mile in 10 min [16]. One minute of vigorous-intensity physical activity equals 2 minutes of moderate-intensity physical activity [16]. There is evidence suggesting that the intensity of physical activity may be a more important determinant of health benefits than frequency or duration [15]. There is a substantial body of evidence comparing the cardiovascular and mortality benefits of various intensities of physical activity and some of the findings have not been entirely consistent. Findings from a recent systematic review suggest that light-intensity physical activity provides similar benefit for all-cause and cardiovascular mortality compared with moderate-intensity physical activity [15]. Though vigorous-intensity physical activity has mostly been shown to be associated with significantly greater risk reductions in adverse cardiovascular outcomes compared with light- or moderate-intensity physical activity [17–22], a pooled analysis of prospective cohorts showed that the greatest mortality benefits were from light-intensity physical activity at doses below physical activity guideline recommendations [23].

**Table 1.** Classification of physical activity intensity.

	Light-intensity	Moderate intensity	Vigorous-intensity
METs	1.1–2.9	3.0–5.9	≥6
Examples	Walking <4.7 km/hr, bicycling < 5 mph, stretching, light weight training, dancing slowly, light yard and housework	Walking at moderate or brisk pace (4.16.5 km/h), slow cycling (15 km/h), painting/decorating, vacuuming, gardening (mowing lawn), golf (pulling clubs in trolley), tennis (doubles), ballroom dancing, water aerobics, weight training	Race-walking, jogging, or running, cycling >15 km/h, heavy gardening (continuous digging or hoeing), swimming laps, tennis (singles), squash or racquetball, rowing, cross-country skiing

Metabolic equivalents (METs) is estimated as the energy cost of a given activity divided by resting energy expenditure: 1 MET = 3.5 3.5 ml of oxygen uptake per kilogram of body weight per minute for the average adult.

The ideal dose of physical activity and/or exercise for optimal health outcomes including cardiovascular health is uncertain [8,9]. However, current physical activity guidelines recommend that adults should engage in a minimum of 150 min of moderate-intensity physical activity or 75 min of vigorous-intensity physical activity per week or an equivalent combination of both types of physical activity per week [8,9,24]; these recommendations are based on the fact that these levels are associated with significant health benefits for the vast majority of individuals and that maximal survival benefit is provided by achieving a physical activity level of 500–1000 MET-min/week [23]. The evidence supporting the cardiovascular health benefits of physical activity and exercise training is well-established. Many studies have presented compelling evidence showing that regular physical activity is associated with reduced risk of adverse cardiovascular outcomes in the general population [25–32].

While the role of physical activity in primary prevention is unequivocal, its significance in secondary prevention (among those with preexisting CVD) is less definitive. Guidelines universally recommend physical activity as part of the secondary preventive strategy. However, the empirical evidence underpinning these recommendations is not as robust. Some studies suggest that the benefits of physical activity are greater for secondary than for primary prevention of CVD [33–35]. Yet, there remains a degree of uncertainty, with limited and sometimes conflicting evidence on the optimal type, intensity, and frequency of exercise required to confer these benefits in patients with established CVD. Considering the profound public health implications of CVD, any intervention that can modify its course is of paramount importance. Individuals with preexisting CVD are particularly vulnerable, often grappling with heightened risks of morbidity and mortality; the risk of a recurrent event is around 50% in the year after a CVD event and up to 75% within 3 years [36]. Furthermore, individuals with preexisting CVD are less likely to engage in physical activity than those without CVD [37–39].

Cardiac rehabilitation (CR), an important and complex tool used in the secondary prevention of CVD, involves the use of a variety of therapies that include education, lifestyle behavior modification, psychosocial support, and supervised exercise programs [40]. Exercise therapy or training is an integral component of CR [41,42]. In the context of exercise-based CR, there are different types of aerobic exercise training that can be differentiated by their intensity and duration. Moderate intensity continuous training (MICT) generally consists of 30–60 min of aerobic exercise at 64–76% peak heart rate [43], while interval training involves more intense bouts interspersed by recovery periods [44]. Interval training can be classified into high-intensity interval training (HIIT) or sprint interval training (SIT) based on intensity. High-intensity interval training involves performing repeated bouts of exercise at an intense effort interspersed by low-intensity exercise or periods of rest with varied recovery times. The exercise periods may range from 5 s to 8 min long with recovery periods varying in length and with total exercise duration lasting between 20 and 60 min [45]. Sprint interval training is defined as supramaximal exertion (e.g. 8 × 20-second intervals at 170% peak work rate) with active recovery/rest between intervals [46]. There is a consistent body of evidence demonstrating that HIIT yields superior health benefits compared to MICT in individuals with preexisting CVD [47–49]. However, outcomes mostly evaluated have included cardiorespiratory fitness (CRF), cardiovascular function, cardiovascular risk factors, and measures of motor performance [47–49]. The evidence for long-term outcomes such as CVD and mortality is sparse and limited.

Given the overall evidence, it is essential to review the existing literature to understand the role of physical activity and/or exercise training in influencing cardiovascular outcomes in individuals with preexisting CVD. This review article aims to bridge the knowledge gap by summarizing available observational and interventional evidence on the relationships between physical activity and/or exercise and adverse cardiovascular outcomes among those with preexisting CVD. Through this exploration, we aim to elucidate the postulated biologic mechanisms, highlight areas of prevailing uncertainty, and discuss potential implications for patient care and public health.

## 2. Methods

A search of MEDLINE and Embase was conducted up to January 2024 for randomized controlled trials (RCTs), non-RCTs and observational studies, including prospective cohort, nested case-control, case-cohort or retrospective cohort studies, with a particular focus on systematic reviews and meta-analyses of these study designs, based on the hierarchy of evidence [50]. Search terms or keywords related to physical activity (‘physical activity,’ ‘exercise’), adverse cardiovascular outcomes (‘cardiovascular disease,’ ‘coronary heart disease,’ ‘stroke,’ ‘heart failure,’ ‘atrial fibrillation,’ ‘mortality’) and preexisting CVD (‘secondary prevention’) were combined. The review was restricted to studies conducted in human populations, reported in English, and in adults. For observational studies, the focus was particularly on longitudinal cohort studies given that they address the issue of temporality. We only included studies that reported on adverse cardiovascular outcomes. Though CRF is a measure of regular aerobic physical activity or exercise, we did not evaluate studies that specifically assessed the effects of CRF on adverse cardiovascular outcomes in individuals with preexisting CVD. However, studies that reported the effects of physical activity and/or exercise training on CRF and reported on adverse cardiovascular outcomes were included. Studies that specifically assessed the combined effects of physical activity and other lifestyle or risk factor management (e.g. diet, lipid and glycemic control, blood pressure reduction) were not included. Given that CR may involve a combination of education, lifestyle behavior modification, psychosocial support, and supervised exercise programs [40], we did not include studies of CR; however, we included studies that specifically evaluated the effects of exercise-based CR, supervised exercise programs or exercise training on adverse cardiovascular outcomes.

## 3. Impact of physical activity and/or exercise training on adverse cardiovascular outcomes in preexisting CVD

### 3.1. Impact of physical activity

Using the Nord-Trøndelag health (HUNT) population-based prospective study comprising 2137 men and 1367 women with CHD followed up for 18 years, Moholdt and colleagues [51] in 2008 assessed the relationships of exercise amount and intensity with the risk of mortality. Compared with no activity, one weekly exercise session was associated with a lower risk of all-cause mortality − 20% risk reduction in men and 32% risk reduction in women. The association was stronger with increasing frequency and those who reported moderate or high-intensity exercise had a somewhat lower risk of death than those who exercised at low intensity [51]. Hamer and Stamatakis [52] in 2009 evaluated associations between different types of physical activity (domestic, walking, sports) and mortality in participants with established CVD using data collected from the Scottish Health Surveys. The results showed that the lowest risks for all-cause mortality were seen in participants undertaking at least 20 min/week of sports activity (68% relative risk reduction) or walking (26% relative risk reduction). The associations were similar for CVD mortality [52]. Among adults post-acute stroke, physical activity assessed via accelerometry was shown to be inversely associated with all-cause mortality [53]. Stewart and colleagues [54] in 2017 analyzed the association between self-reported volume of habitual exercise and mortality in 15,486 patients from 39 countries with stable CHD who participated in the Stabilization of Atherosclerotic Plaque by Initiation of Darapladib Therapy (STABILITY) study. The results showed a gradual decrease in risk of all-cause and CVD mortality with a gradual increase in self-reported habitual exercise, but these were not consistent with linear dose-response relationships. The largest benefits occurred between sedentary patient groups and between those with the highest mortality risk [54]. Jeong and colleagues [34] in 2019 compared the impact of leisure-time physical activity on mortality in primary versus secondary cardiovascular prevention using a population-based cohort of 131,558 and 310,240 participants with and without CVD, respectively. The authors demonstrated an inverse relationship between physical activity level and the mortality risk in both groups. The benefit in the secondary prevention group was greater than that in the primary prevention group; every 500 MET-min/week increase in physical activity resulted in a 14% and 7% risk reduction in mortality in the secondary and primary prevention groups, respectively (*p*-value for interaction < 0.001). In addition, while individuals without CVD benefited the most between 1 and 500 MET-min/week of physical activity, the benefit in those with CVD continued above 500–1000 MET-min/week [34]. Using an international prospective registry of 32,370 consecutive outpatients with stable coronary artery disease who were followed for up to five years, Biscaglia and colleagues [55] in 2020 ascertained the relationship between physical activity levels and cardiovascular outcomes. Their results showed nonlinear relationships between frequency and intensity of physical activity and lower risk of cardiovascular outcomes. Comparing vigorous physical activity once or twice per week with no or a low level of physical activity was associated with an 18% lower risk of the composite of cardiovascular death, myocardial infarction (MI) and stroke. Engaging in more frequent exercise did not result in further outcome benefit [55]. Bakker and colleagues [56] in 2021 compared the associations between moderate to vigorous physical activity (MVPA) and incident major adverse cardiovascular events (MACE) and mortality between healthy individuals, individuals with elevated levels of cardiovascular risk factors (CVRF), and CVD using a cohort study of 142,493 participants. The authors observed that MVPA was beneficial for reducing adverse cardiovascular outcomes, but the shape of the association was dependent on cardiovascular health status. A curvilinear association was found in healthy and CVRF individuals with a steep risk reduction at low to moderate MVPA volumes and benefits plateauing at higher MVPA volumes. However, there was a linear association in participants with preexisting CVD, suggesting a constant risk reduction with higher volumes of MVPA. The authors concluded that individuals with preexisting CVDs should be encouraged that ‘more is better’ regarding MVPA [56]. Kim and colleagues [33] in 2022 utilizing the Korean National Health Insurance Service database comprising 68,223 participants, investigated the effect of physical activity on mortality in elderly populations with or without CVD. Their principal findings showed that physical activity was associated with a reduced risk of all-cause mortality in older adults with or without CVD, and the benefits of physical activity were greater in patients with CVD than in those without CVD. Additionally, physical activity was associated with a reduced risk of all-cause mortality in older adults irrespective of the specific underlying CVD; the risk of mortality progressively decreased with increasing physical activity in patients with heart failure (HF) or MI but reached a plateau in patients with stroke or peripheral artery disease. Finally, the benefits of physical activity were greater in patients with stroke or HF [33]. Using a large population-based cohort of US adults, Cabanas-Sánchez and colleagues [35] in 2023 assessed the association of physical activity with cause-specific CVD mortality among people with preexisting CVD and compared this with the relationship of physical activity with CVD-related mortality in people without CVD. In their fully adjusted models, compared with no physical activity, recommended and additional physical activity was associated with 37.1% and 42.0% lower risk of CVD mortality among people with prior CHD, respectively. Among people with stroke, recommended and additional physical activity was related to 30.7% and 59.3% lower risk of cerebrovascular mortality, respectively. The protective effect of physical activity on cause-specific cardiovascular mortality was greater in people with CVD than in those without prior CVD [35]. Additionally, the authors observed an inverse nonlinear dose-response relationship between physical activity and cause-specific cardiovascular mortality in all populations that were studied [35].

Though there is a complex dose-response relationship between physical activity, exercise and AF, most of the evidence suggest primary prevention benefits based on guideline recommended physical activity levels [57]. There is also emerging evidence on the role of physical activity and/or exercise training in the secondary prevention of AF or adverse cardiovascular outcomes in individuals with preexisting AF. Pathak and colleagues [58] in 2015 evaluated the role of CRF (via exercise training) on AF recurrence in obese individuals with AF and showed that improved CRF was associated with decreased AF recurrence. Proietti and colleagues [59] in 2017 evaluated the relationship between self-reported physical activity and major adverse outcomes in patients with AF using the EURObservational Research Programme on AF (EORP-AF) Pilot Survey. They observed that regular exercise and intense physical activity was associated with a lower risk of CVD death, all-cause mortality and thromboembolic events irrespective of sex, age, AF symptomatic status, or risk of stroke [59]. In 1117 AF patients from the HUNT3 study, Garnvik and colleagues [60] in 2020 examined the prospective associations of physical activity and estimated CRF with adverse cardiovascular outcomes. Individuals meeting recommended physical activity guidelines had 45% lower risk of all-cause and 46% lower risk of CVD mortality compared with inactive individuals. The respective risk reductions for CVD morbidity and stroke were 22% and 30%. Malmo and colleagues [61] using a RCT conducted in 51 patients with nonpermanent AF, demonstrated that 12 weeks of aerobic interval training significantly reduced AF burden from 8.1 to 4.8%, with no significant change in the non-exercise control group.

### 3.2. Impact of exercise training

In a multicenter, RCT of 2331 medically stable outpatients with HF and reduced ejection fraction randomized to usual care plus aerobic exercise training versus usual care alone, O’Connor and colleagues in 2009 showed that exercise training was associated with modest significant reductions for both all-cause mortality or hospitalization and cardiovascular mortality or HF hospitalization [62].

A 2011 systematic review and meta-analysis of 34 RCTs (follow-up of 3 months-5 years) assessed the efficacy of exercise-based CR following MI and demonstrated a lower risk of re-infarction (47% relative risk reduction), cardiac mortality (36% relative risk reduction), and all-cause mortality (26% relative risk reduction) in patients randomized to exercise-based CR [63]. In a 2016 Cochrane systematic review and meta-analysis of exercise-based CR following CHD (MI or revascularization), analysis of 63 RCTs (median follow-up of 12 months) showed that exercise-based CR vs no-exercise controls led to a reduction in cardiovascular mortality (26% relative risk reduction) and the risk of hospital admissions (18% relative risk reduction), with no significant effect on all-cause mortality, MI, or revascularization [40]. An updated 2021 Cochrane Review comprising 85 RCTs supported the conclusions of the previous version; exercise-based CR vs no-exercise controls reduced the risk of MI and provided a modest reduction in all-cause mortality and a large reduction in all-cause hospitalization [64]. In addition, there were improved healthcare costs and health-related quality of life up to 12 months’ follow-up [64]. In a 2021 meta-analysis of 22 RCTs that assessed the effects of exercise therapy compared with no exercise control in patients with CHD, exercise therapy reduced all-cause hospital admissions (54% relative risk reduction) and cardiovascular mortality (56% relative risk reduction), with no significant effect on cardiovascular hospital admissions, all-cause mortality, incidence of MI, or revascularization [65].

## 4. Potential adverse cardiovascular events of physical activity in preexisting CVD

There have been previous indications that vigorous-intensity physical activity might trigger adverse cardiovascular events such as arrhythmias, arrhythmogenic and pathologic cardiac remodeling, AF, MI or sudden cardiac death, particularly for athletes who engage in endurance activities or individuals with existing conditions such as hypertrophic cardiomyopathy or Brugada Syndrome [66–71]. Indeed, the intensities and volumes of these vigorous-intensity physical activity regimens far exceed guideline recommendations. Indications of an increased risk of adverse cardiovascular outcomes or decreased cardiovascular benefits associated with physical activity levels that exceed moderate-intensity physical activity have been observed in the general population [23,72–74] and the risk might be greater in those with preexisting CVD. Heidbüchel and colleagues [75] in 2003 reported on 46 high-level endurance athletes with ventricular arrhythmias who were followed-up for a median of 4.7 years; 18 of them developed a major arrhythmic event, of which 9 were sudden deaths. Batty and colleagues [76] in 2003 also analyzed data from a 25 year follow-up of mortality for 6474 male British civil servants who underwent a resting electrocardiogram (ECG) and responded to queries regarding angina at study entry. Among men who had ECG abnormalities, physical activity was associated with a higher rate of CHD mortality [76]. In a prospective cohort of 1038 participants with stable CHD in which frequency of strenuous leisure time physical activity was assessed repeatedly over 10 years of follow-up, Mons and colleagues in 2014 showed a reverse J-shaped relationship between physical activity level and cardiovascular mortality, with the most frequently active patients also having an increased risk [77]. In a large cohort of 2377 self-identified heart attack survivors, Williams and Thompson in 2014 showed that running or walking decreased CVD mortality risk progressively at most levels of exercise, but the risk of CVD mortality and ischemic heart disease deaths was increased at the highest levels of exercise (running: above 7.1 km/d or walking briskly: 10.7 km/d) [78]. In the study by Stewart and colleagues in 2017 [54], the authors acknowledged that a modest increase in mortality associated with high intensities or durations of vigorous exercise could not be excluded, given that the study’s long durations of vigorous physical activity may not have been accurately quantified and the 95% confidence intervals for the risk estimates were wide.

## 5. Postulated mechanisms underlying the beneficial and adverse cardiovascular effects of physical activity

**Figure 1.**
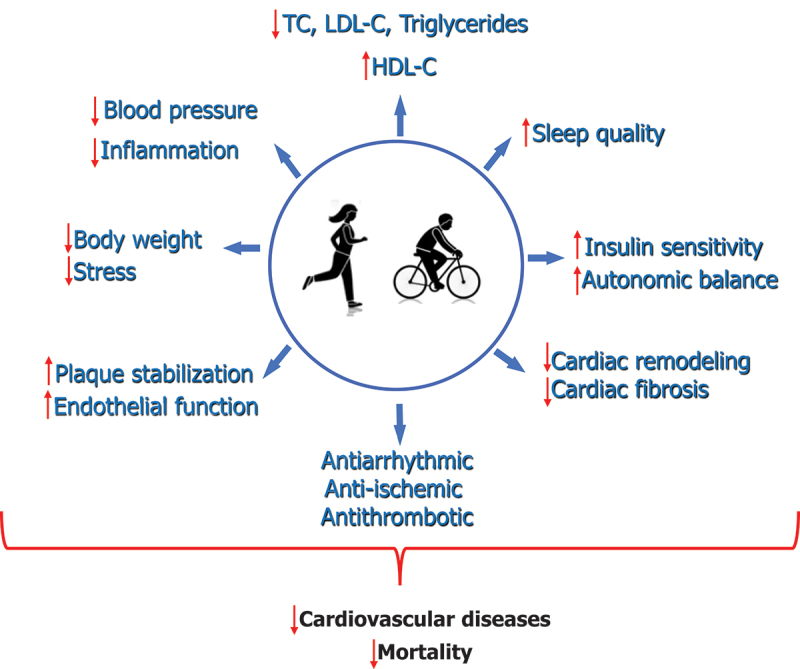
Proposed mechanistic pathways underlying the associations between regular physical activity and reduced risk of adverse cardiovascular outcomes.

HDL-C, high-density lipoprotein cholesterol; LDL-C, low-density lipoprotein cholesterol; TC, total cholesterol.

Physical activity plays a pivotal role in promoting cardiovascular health, particularly among those with preexisting CVD. Various mechanistic pathways are proposed to elucidate this protective role, highlighting the multi-faceted benefits of regular physical activity or exercise (Figure 1). Here, we review several of these mechanisms, as well as postulate reasons for the enhanced benefits observed in those with established CVD. Physical activity has been consistently shown to beneficially alter the levels of several conventional risk factors associated with CVD. These include reductions in resting blood pressure and positive modifications in lipid profiles and body weight [32,79–81]. Furthermore, it assists in better glucose profiles, reducing levels of inflammatory markers, and improving insulin sensitivity [32,82,83]. Exercise exerts various direct cardioprotective effects beyond traditional risk factors, including antiarrhythmic, anti-ischemic, and antithrombotic actions as well as improving autonomic nervous system (ANS) balance [84,85]. Physical activity has been shown to enhance the function of the endothelium [86,87], thereby decreasing the risk of atherosclerosis formation, which improves vascular health and reduces the risk of CVD complications. Regular physical activity might promote the stabilization of atherosclerotic plaques [88], thereby reducing the risk of plaque rupture and subsequent cardiovascular events such as MI. Chronic heart conditions can cause detrimental changes in the cardiac structure, a process termed cardiac remodeling; physical activity can reverse or attenuate some of these changes [89], thereby preserving or enhancing cardiac systolic and diastolic function. Exercise can also cause attenuation in cardiac fibrosis, promoting better cardiac muscle health [90]. Regular physical activity can lead to better sleep quality and reduced perceived stress via improvement in ANS balance [32,91,92], both of which have been linked to improved cardiovascular outcomes [93,94]. An intriguing hypothesis suggests that individuals with preexisting CVD might experience more pronounced benefits from exercise due to the synergistic effects of their CVD medications [95]. Many medications used to treat CVD may work in tandem with exercise, magnifying the beneficial adaptations to physical activity. In conclusion, the beneficial effects of physical activity in individuals with preexisting CVD are multifactorial, encompassing direct cardiovascular effects, systemic benefits, and potential interactions with therapeutic interventions.

Vigorous-intensity physical activity or intense exercise may trigger adverse cardiovascular events such as AF, ventricular arrhythmias and sudden deaths in individuals with preexisting CVD via the following pathways: (i) arrhythmogenic and pathologic cardiac remodeling including adverse right ventricle remodeling [67]; (ii) elevated levels of cardiac biomarkers such as troponin and B-type natriuretic peptides [96], which are also known cardiovascular risk factors; and (iii) least physically active individuals who perform unaccustomed physical activity [97].

## 6. Conclusions

In summary, the comprehensive analysis of existing observational and interventional evidence underscores the profound impact of regular physical activity and/or exercise training on mitigating adverse cardiovascular outcomes in individuals with preexisting CVD. Several key findings emerge from the body of research. The evidence consistently demonstrates that individuals with preexisting CVD who engage in regular physical activity experience a notable reduction in the risk of adverse cardiovascular outcomes. This reduction provides compelling support for the integration of physical activity into the management and prevention strategies for CVD. Remarkably, those with preexisting CVD seem to derive even greater benefits from physical activity than their counterparts without CVD, when engaging at the same levels of physical activity. This finding underscores the importance of tailored exercise interventions for this population. The beneficial effects of physical activity were irrespective of age and gender, thereby making it a universally applicable therapeutic modality for individuals with preexisting CVD. However, it is worth noting that women have lower rates of physical activity than men given that women are more subject to disparities arising from sociocultural, sociocultural and political factors [98,99]. Furthermore, this disparity is more prevalent in countries where social, cultural or religious norms restrict women from participating in physical activity [98]. Another notable finding was the significant variation in physical activity responses among specific cardiovascular conditions; for instance, individuals with cardiovascular conditions such as stroke or HF may experience even more substantial advantages from regular physical activity. The dose-response relationships between the intensity, frequency, duration and volume of physical activity and adverse cardiovascular outcomes in individuals with preexisting CVD are complex. While a general trend suggests that more physical activity leads to better outcomes, some studies hint at an increased risk of adverse cardiovascular events at extremely high levels of physical activity. Nevertheless, it’s essential to emphasize that these extreme levels typically surpass recommended guidelines by a significant margin. The evidence seems to suggest that a physical activity level of 500 MET-min/week (which is equivalent to the minimal guideline recommendation i.e. 150 min of moderate-intensity physical activity or 75 min of vigorous-intensity physical activity or an equivalent combination) may be a minimum requirement for patients with CVD. Currently, guideline recommendations on the level of physical activity for secondary prevention in specific CVD groups are not very clear and also not consistent. For instance, the 2019 European Society of Cardiology (ESC) guidelines recommend 30–60 min of moderate-intensity aerobic activity ≥5 days per week for patients with chronic coronary syndromes (CCS) [100]. The 2020 ESC Guidelines on sports cardiology and exercise in patients with CVD [101] recommend (i) risk stratification for exercise-induced adverse events in individuals with established (long-standing) CCS prior to engaging in exercise and (ii) regular discussion about exercise participation and provision of an individualized exercise prescription in all individuals with HF. The 2023 AHA/ACC/ACCP/ASPC/NLA/PCNA Guideline for the Management of Patients With Chronic Coronary Disease recommends an exercise regimen including ≥150 minutes/week of moderate-intensity aerobic activities or ≥75 minutes/week of higher-intensity aerobic activities for patients with chronic coronary disease who do not have contraindications [102]; these physical activity levels are similar to that recommended for healthy adults [8,9,24]. Perhaps one of the most striking findings in this synthesis is that sedentary individuals stand to gain the most from becoming physically active. The benefits are most pronounced when transitioning from a sedentary lifestyle to low-to-moderate levels of habitual exercise. As individuals progress from higher baseline fitness levels, the incremental gains become less substantial. Finally, the review showed consistent reductions in adverse cardiovascular outcomes following exercise-based CR or structured exercise training in individuals with preexisting CVD. These findings reflect guidelines by bodies such as the ESC which recommend that patients with cardiac conditions should participate in a medically supervised, structured and exercise-based CR program, which should start as soon as possible after the initial cardiac event [103,104].

## 7. Expert opinion

These findings have profound implications for both clinical practice and public health policy. One of the most salient observations from this comprehensive analysis is the need for tailored exercise interventions in individuals with preexisting CVD. Healthcare providers and guideline bodies should recognize that physical activity prescriptions should not follow a ‘one-size-fits-all’ approach. To reap the maximum benefits of physical activity and minimize adverse effects, physical activity and/or exercise regimens should be tailored to unique factors such as an individual’s baseline physical activity habits, cardiovascular health status and the specific nature of their CVD. Whether it’s addressing the challenges of HF, stroke, or other cardiac conditions, an individualized exercise regimen holds tremendous promise. Public health initiatives should prioritize the promotion of physical activity as a cornerstone of CVD prevention and management. Clinicians should consider exercise as a prescription in its own right, which should be integrated seamlessly into the continuum of care for patients with CVD. The complex dose-response relationships explored in this review emphasize the need for a carefully balanced approach to exercise prescription. While a general trend suggests that more physical activity leads to better outcomes, caution is warranted at extreme levels. Striking the right balance is essential. The identified threshold of 500 MET-min/week as a minimum requirement for patients with CVD, equivalent to guideline recommendations, provides a concrete target for clinicians and patients alike. Raising awareness about the benefits of regular exercise, particularly among sedentary individuals and those with CVD, can significantly impact population health. Incorporating physical activity as an integral component of cardiovascular care plans can lead to more favorable outcomes and reduce the burden of CVD. Clinicians should actively engage patients in discussions about physical activity, encouraging them to adopt sustainable exercise routines.

Despite the clinical effectiveness and cost benefits of CR as well as the existence of guideline recommendations, the uptake rates and average duration of CR are still suboptimal [105]. The following have been identified as barriers to the implementation and use of CR: older age, low socioeconomic status and educational level, lack of awareness on its benefits, presence of comorbidities, financial issues plus transportation problems, lack of automatic referral, no financial incentives, lack of multidisciplinary teams, time consuming, reimbursement issues, lack of specialized locations, and geographical issues [105]. The following strategies may improve access to and uptake of CR: (1) public education campaigns should emphasize the importance of CR after cardiac events, targeting not only patients but also healthcare providers; (2) implementing automatic referral systems within healthcare institutions can significantly increase CR utilization; (3) addressing financial barriers can involve providing subsidies or insurance coverage for CR sessions; (4) designing CR programs that cater to the specific needs of older adults and patients with comorbidities is essential; (5) expanding the availability of virtual CR programs can overcome geographical issues and time constraints; (6) considering offering incentives such as discounts on health insurance premiums or rewards for program completion; (7) establishing CR programs in community centers and local facilities, making them more accessible and reducing the need for specialized locations; (8) promoting a culture of prevention in healthcare, emphasizing the importance of CR as a preventative measure to reduce future cardiovascular events; and (9) advocacy efforts should aim to influence policy changes that mandate automatic referral, insurance coverage, and reimbursement for CR services.

In the absence of specific guideline recommendations for specific CVD populations, walking is a simple but powerful cardioprotective physical activity that should be encouraged among these populations who don’t have mobility problems. Walking requires no special facilities and can be done at any time of day, making it an ideal form of physical activity for most people [32]. There is little evidence to suggest that walking increases the risk of injuries or serious adverse events. A brisk walk for at least 30 minutes, 5 days a week, meets the current physical activity guidelines [8,9,24].

### 7.1. Future research directions

Looking ahead, several areas warrant further exploration. Though the threshold of 500 MET-min/week seems to be the minimum physical activity or exercise requirements for individuals with CVD, the optimal ideal dose suitable for all individuals with preexisting CVD is still yet to be identified. Future research should delve into the concept of individualized exercise prescriptions; studies should explore the physical activity doses that may be suitable for specific cardiovascular conditions as well as taking into consideration different patient factors such as baseline physical activity status and cardiovascular health status. Longitudinal studies with objective measures of physical activity are needed to better understand the dose-response nature of the relationships, particularly the long-term effects of sustained physical activity in individuals with preexisting CVD and specific types of CVD. Studies which track patients over long follow-up periods can provide insights into the durability of benefits and the potential for disease progression. Additionally, investigations into strategies that can maintain or increase exercise adherence over the long term are warranted. As our understanding of the dose-response relationship between physical activity and adverse cardiovascular outcomes evolves, there is a need to define optimal exercise parameters with greater precision. Research should aim to establish clear guidelines for exercise intensity, duration, frequency, and mode, considering the specific needs of individuals with diverse cardiovascular conditions. Future research should also aim to address the gender disparities in physical activity. These could include (1) investigating culturally tailored interventions that respect and accommodate cultural norms and values; (2) evaluating the effectiveness of community-based physical activity programs specifically designed for women; (3) studying the impact of educational campaigns aimed at increasing awareness about the importance of physical activity for women’s cardiovascular health; (4) exploring gender-responsive approaches in healthcare settings that consider women’s unique needs and preferences regarding physical activity; (5) evaluating the effectiveness of smartphone apps and wearable devices tailored to women’s needs; (6) investigating the impact of policy changes that promote gender equity in access to physical activity opportunities; (7) evaluating the role of peer support networks in encouraging physical activity; (8) examining strategies to promote long-term adherence to physical activity among women; and (9) investigating the influence of family and social support systems on women’s physical activity.

Investigating the underlying mechanisms by which physical activity exerts its cardiovascular benefits such as its impact of exercise on inflammation, endothelial function, oxidative stress, and cardiac remodeling, among others, can unveil novel therapeutic targets and interventions. There should be a special focus on vulnerable populations, including older adults, individuals with multiple comorbidities, and underserved communities. Tailored exercise interventions that account for the unique challenges and needs of these groups can significantly impact health disparities in cardiovascular outcomes. A comprehensive understanding of the economic and healthcare system implications of promoting physical activity is also essential. Research should assess the cost-effectiveness of exercise-based interventions and their potential to reduce the overall healthcare burden associated with CVD.

Future research directions for CR should aim to enhance the effectiveness, accessibility, and inclusivity of CR, which will ultimately improve outcomes for individuals with preexisting CVD. These could include (1) exploring the effectiveness of virtual CR programs; (2) evaluating the long-term outcomes of CR; (3) focusing on improving access to CR for underserved populations and reducing disparities in participation rates; (4) determining the optimal timing for initiating CR after a cardiovascular event or procedure is essential; (5) exploring the psychosocial aspects of CR, such as mental health support, stress management, and motivation enhancement; and (6) investigating novel interventions beyond traditional exercise, can expand the scope of CR research.

In conclusion, the body of evidence unequivocally supports the pivotal role of physical activity and exercise training in reducing adverse cardiovascular outcomes in individuals with preexisting CVD. These findings should serve as a call for clinicians, public health officials, and researchers to collaborate in advancing strategies that harness the potential of physical activity to enhance cardiovascular health and well-being, recognizing the diverse needs of each patient and the complexity of the dose-response relationship.

## Article highlights

- Consistent evidence shows that individuals with preexisting CVD who engage in regular physical activity experience a notable reduction in the risk of adverse cardiovascular outcomes, with significant variations in physical activity responses among specific cardiovascular conditions.
- Individuals with preexisting CVD seem to derive even greater benefits from physical activity than their counterparts without CVD, when engaging at the same levels of physical activity.
- The dose-response relationships between the intensity, frequency, duration and volume of physical activity and adverse cardiovascular outcomes in individuals with preexisting CVD are complex; however, though a general trend suggests that more physical activity leads to better outcomes, some evidence suggests an increased risk of adverse cardiovascular events at extremely high levels of physical activity.
- A striking finding is that sedentary individuals stand to gain the most from becoming physically active.
- A physical activity level of 500 MET-min/week (equivalent to 150 min of moderate-intensity physical activity or 75 min of vigorous-intensity physical activity or an equivalent combination) may be a minimum requirement for patients with preexisting CVD.
- There were consistent reductions in adverse cardiovascular outcomes following exercise-based CR or structured exercise training in individuals with preexisting CVD.

## References

[1] Mensah GA, Fuster V, Murray CJL, et al. Global Burden of Cardiovascular Diseases and Risks, 1990-2022. J Am Coll Cardiol. 2023 Dec 19;82(25):2350–2473. doi: 10.1016/j.jacc.2023.11.007 38092509 PMC7615984

[2] Mensah GA, Roth GA, Fuster V. The global burden of cardiovascular diseases and risk factors: 2020 and beyond. J Am Coll Cardiol. 2019 Nov 19;74(20):2529–2532. 31727292 10.1016/j.jacc.2019.10.009

[3] Roth GA, Mensah GA, Fuster V. The global burden of cardiovascular diseases and risks: a compass for global action. J Am Coll Cardiol. 2020 Dec 22;76(25):2980–2981. 33309174 10.1016/j.jacc.2020.11.021

[4] Vaduganathan M, Mensah GA, Turco JV, et al. The global burden of cardiovascular diseases and risk: a compass for future health. J Am Coll Cardiol. 2022 Dec 20;80(25):2361–2371. doi: 10.1016/j.jacc.2022.11.005 36368511

[5] Lloyd-Jones DM, Hong Y, Labarthe D, et al. Defining and setting national goals for cardiovascular health promotion and disease reduction: the American Heart Association’s strategic impact goal through 2020 and beyond. Circulation. 2010 Feb 2;121(4):586–613. doi: 10.1161/CIRCULATIONAHA.109.192703 20089546

[6] Lloyd-Jones DM, Allen NB, Anderson CAM, et al. Life’s essential 8: updating and enhancing the American Heart Association’s Construct of Cardiovascular Health: a Presidential Advisory from the American Heart Association. Circulation. 2022 Aug 2;146(5):e18–e43. doi: 10.1161/CIR.0000000000001078 35766027 PMC10503546

[7] World Health Organization. The global action plan on physical activity 2018 - 2030. [cited 2023 Sep 29]. Available from: https://www.who.int/news-room/initiatives/gappa/action-plan

[8] Bull FC, Al-Ansari SS, Biddle S, et al. World Health Organization 2020 guidelines on physical activity and sedentary behaviour. Br J Sports Med. 2020 Dec;54(24):1451–1462. doi: 10.1136/bjsports-2020-102955 33239350 PMC7719906

[9] Piercy KL, Troiano RP, Ballard RM, et al. The physical activity guidelines for americans. JAMA. 2018 Nov 20;320(19):2020–2028. 10.1001/jama.2018.14854 30418471 PMC9582631

[10] Sedentary Behaviour Research N. Letter to the editor: standardized use of the terms “sedentary” and “sedentary behaviours”. Appl Physiol Nutr Metab. 2012, Jun;37(3):540–542. doi: 10.1139/h2012-024 22540258

[11] Tremblay MS, Aubert S, Barnes JD, et al. Sedentary Behavior Research Network (SBRN) - terminology consensus project process and outcome. Int J Behav Nutr Phys Act. 2017 Jun 10;14(1):75. doi: 10.1186/s12966-017-0525-8 28599680 PMC5466781

[12] American College of Sports Medicine. ACSM’s guidelines for exercise testing and prescription. 10th ed. Philadelphia (PA): Lippincott Williams & Wilkins; 2017.

[13] Blair SN, Kohl HW, Gordon NF, et al. How much physical activity is good for health? Annu Rev Public Health. 1992;13(1):99–126. doi: 10.1146/annurev.pu.13.050192.000531 1599603

[14] Strath SJ, Kaminsky LA, Ainsworth BE, et al. Guide to the assessment of physical activity: clinical and research applications: a scientific statement from the American Heart Association. Circulation. 2013 Nov 12;128(20):2259–79. doi: 10.1161/01.cir.0000435708.67487.da 24126387

[15] Moxley E, Habtzghi D. A systematic review comparing dose response of exercise on cardiovascular and all-cause mortality. Home Health Care Manag Pract. 2019;31(4):263–273. doi: 10.1177/1084822319831929

[16] Physical Activity Guidelines Advisory Committee. Physical activity guidelines advisory committee report, 2008 (pp. A1-H14). WA (DC): US Department of Health and Human Services; 2008. 10.1111/j.1753-4887.2008.00136.x 19178654

[17] Lee IM, Paffenbarger RS Jr. Associations of light, moderate, and vigorous intensity physical activity with longevity. The harvard alumni health study. Am J Epidemiol. 2000 Feb 01;151(3):293–299. 10670554 10.1093/oxfordjournals.aje.a010205

[18] Tanasescu M, Leitzmann MF, Rimm EB, et al. Exercise type and intensity in relation to coronary heart disease in men. JAMA. 2002 Oct 23-30;288(16):1994–2000. doi: 10.1001/jama.288.16.1994 12387651

[19] Chomistek AK, Cook NR, Flint AJ, et al. Vigorous-intensity leisure-time physical activity and risk of major chronic disease in men. Med Sci Sports Exerc. 2012 Oct;44(10):1898–905. 22543741 10.1249/MSS.0b013e31825a68f3 PMC3445709

[20] Williams PT. Reductions in incident coronary heart disease risk above guideline physical activity levels in men. Atherosclerosis. 2010 Apr;209(2):524–7. doi: 10.1016/j.atherosclerosis.2009.09.018 19815208 PMC3776591

[21] Williams PT. Reduced total and cause-specific mortality from walking and running in diabetes. Med Sci Sports Exerc. 2014;46(5):933–9. doi: 10.1249/MSS.0000000000000197 24968127 PMC4157907

[22] Powell KE, Paluch AE, Blair SN. Physical activity for health: what kind? How much? How intense? On top of what? Annu Rev Public Health. 2011;32(1):349–65. doi: 10.1146/annurev-publhealth-031210-101151 21128761

[23] Arem H, Moore SC, Patel A, et al. Leisure time physical activity and mortality: a detailed pooled analysis of the dose-response relationship. JAMA Intern Med. 2015 Jun;175(6):959–67. 25844730 10.1001/jamainternmed.2015.0533 PMC4451435

[24] UK Chief Medical Officers’ Physical Activity Guidelines 2019. [cited 2022 Nov 25]. Available from: https://assets.publishing.service.gov.uk/government/uploads/system/uploads/attachment_data/file/832868/uk-chief-medical-officers-physical-activity-guidelines.pdf

[25] Cheng W, Zhang Z, Cheng W, et al. Associations of leisure-time physical activity with cardiovascular mortality: a systematic review and meta-analysis of 44 prospective cohort studies. Eur J Prev Cardiol. 2018 Nov;25(17):1864–1872. 30157685 10.1177/2047487318795194

[26] Lear SA, Hu W, Rangarajan S, et al. The effect of physical activity on mortality and cardiovascular disease in 130 000 people from 17 high-income, middle-income, and low-income countries: the PURE study. Lancet. 2017 Dec 16;390(10113):2643–2654. doi: 10.1016/S0140-6736(17)31634-3 28943267

[27] Kyu HH, Bachman VF, Alexander LT, et al. Physical activity and risk of breast cancer, colon cancer, diabetes, ischemic heart disease, and ischemic stroke events: systematic review and dose-response meta-analysis for the global burden of disease study 2013. BMJ. 2016 Aug 9;354:i3857. 27510511 10.1136/bmj.i3857 PMC4979358

[28] Gebel K, Ding D, Chey T, et al. Effect of moderate to vigorous physical activity on all-cause mortality in Middle-aged and Older Australians. JAMA Intern Med. 2015 Jun;175(6):970–7. 25844882 10.1001/jamainternmed.2015.0541

[29] Kunutsor SK, Jae SY, Laukkanen JA. ‘Weekend warrior’ and regularly active physical activity patterns confer similar cardiovascular and mortality benefits: a systematic meta-analysis. Eur J Prev Cardiol. 2023 Feb 14;30(3):e7–e10. 36315020 10.1093/eurjpc/zwac246

[30] Kunutsor SK, Makikallio TH, Seidu S, et al. Physical activity and risk of venous thromboembolism: systematic review and meta-analysis of prospective cohort studies. Eur J Epidemiol. 2020 May;35(5):431–442. 31728878 10.1007/s10654-019-00579-2 PMC7250794

[31] Kunutsor SK, Seidu S, Makikallio TH, et al. Physical activity and risk of atrial fibrillation in the general population: meta-analysis of 23 cohort studies involving about 2 million participants. Eur J Epidemiol. 2021 Mar;36(3):259–274. 33492548 10.1007/s10654-020-00714-4 PMC8032592

[32] Ungvari Z, Fazekas-Pongor V, Csiszar A, et al. The multifaceted benefits of walking for healthy aging: from blue zones to molecular mechanisms. Geroscience. 2023 Jul 26;45(6):3211–3239. doi: 10.1007/s11357-023-00873-8 37495893 PMC10643563

[33] Kim MH, Sung JH, Jin MN, et al. Impact of physical activity on all-cause mortality according to Specific cardiovascular disease. Front Cardiovasc Med. 2022;9:811058. doi: 10.3389/fcvm.2022.811058 35187126 PMC8855984

[34] Jeong SW, Kim SH, Kang SH, et al. Mortality reduction with physical activity in patients with and without cardiovascular disease. Eur Heart J. 2019 Nov 14;40(43):3547–3555. doi: 10.1093/eurheartj/ehz564 31504416 PMC6855138

[35] Cabanas-Sanchez V, Duarte Junior MA, Lavie CJ, et al. Physical activity and cause-specific cardiovascular mortality among people with and without cardiovascular disease: a cohort study of 0.6 million US adults. Mayo Clin Proc. 2023 Sep 5; doi: 10.1016/j.mayocp.2023.05.028 37676199

[36] Bansilal S, Castellano JM, Fuster V. Global burden of CVD: focus on secondary prevention of cardiovascular disease. International Journal Of Cardiology. 2015 Dec;201(Suppl 1):S1–7. doi: 10.1016/S0167-5273(15)31026-3 26747389

[37] Vasankari V, Husu P, Vaha-Ypya H, et al. Subjects with cardiovascular disease or high disease risk are more sedentary and less active than their healthy peers. BMJ Open Sport Exerc Med. 2018;4(1):e000363. doi: 10.1136/bmjsem-2018-000363 PMC5950643 29765701

[38] Cassidy S, Fuller H, Chau J, et al. Accelerometer-derived physical activity in those with cardio-metabolic disease compared to healthy adults: a UK biobank study of 52,556 participants. Acta Diabetol. 2018 Sep;55(9):975–979. 29808390 10.1007/s00592-018-1161-8 PMC6096713

[39] Zhao G, Ford ES, Li C, et al. Are United States adults with coronary heart disease meeting physical activity recommendations? Am J Cardiol. 2008 Mar 1;101(5):557–561. doi: 10.1016/j.amjcard.2007.10.015 18307998

[40] Anderson L, Oldridge N, Thompson DR, et al. Exercise-based cardiac rehabilitation for coronary heart disease: Cochrane Systematic Review and meta-analysis. J Am Coll Cardiol. 2016 Jan 5;67(1):1–12. doi: 10.1016/j.jacc.2015.10.044 26764059

[41] Balady GJ, Ades PA, Bittner VA, et al. Referral, enrollment, and delivery of cardiac rehabilitation/secondary prevention programs at clinical centers and beyond: a presidential advisory from the American heart association. Circulation. 2011 Dec 20;124(25):2951–60. doi: 10.1161/CIR.0b013e31823b21e2 22082676

[42] Perk J, De Backer G, Gohlke H, et al. European guidelines on cardiovascular disease prevention in clinical practice (version 2012). The fifth joint task force of the European Society of Cardiology and Other Societies on cardiovascular disease prevention in clinical practice (constituted by representatives of nine societies and by invited experts). Eur Heart J. 2012 Jul;33(13):1635–701. 22555213 10.1093/eurheartj/ehs092

[43] American College of Sports Medicine. ACSM’s guidelines for exercise testing and prescription. 10th ed. Philadelphia: Wolters Kluwer; 2017.

[44] Weston KS, Wisloff U, Coombes JS. High-intensity interval training in patients with lifestyle-induced cardiometabolic disease: a systematic review and meta-analysis. Br J Sports Med. 2014 Aug;48(16):1227–34. doi: 10.1136/bjsports-2013-092576 24144531

[45] American College of Sports Medicine. ACSM’s guidelines for exercise testing and prescription. 9th ed. Philadelphia: Lippincott Williams & Wilkins; 2013. 10.1249/JSR.0b013e31829a68cf 23851406

[46] Williams CJ, Gurd BJ, Bonafiglia JT, et al. A multi-center comparison of O(2peak) trainability between interval training and moderate intensity continuous training. Front Physiol. 2019;10:19. doi: 10.3389/fphys.2019.00019 30804794 PMC6370746

[47] Yue T, Wang Y, Liu H, et al. Effects of high-intensity interval vs. Moderate-intensity continuous training on cardiac rehabilitation in patients with cardiovascular disease: a systematic review and meta-analysis. Front Cardiovasc Med. 2022;9:845225. doi: 10.3389/fcvm.2022.845225 35282360 PMC8904881

[48] Hannan AL, Hing W, Simas V, et al. High-intensity interval training versus moderate-intensity continuous training within cardiac rehabilitation: a systematic review and meta-analysis. Open Access J Sports Med. 2018;9:1–17. doi: 10.2147/OAJSM.S150596 29416382 PMC5790162

[49] Liou K, Ho S, Fildes J, et al. High intensity interval versus moderate intensity continuous training in patients with coronary artery disease: a meta-analysis of physiological and clinical parameters. Heart Lung Circ. 2016 Feb;25(2):166–74. 26375499 10.1016/j.hlc.2015.06.828

[50] OCEBM Levels of Evidence Working Group. The oxford 2011 levels of evidence. Oxford Centre for Evidence-Based Medicine. [cited 2020 Oct 8]. Available from: https://www.cebm.ox.ac.uk/resources/levels-of-evidence/ocebm-levels-of-evidence

[51] Moholdt T, Wisloff U, Nilsen TI, et al. Physical activity and mortality in men and women with coronary heart disease: a prospective population-based cohort study in Norway (the HUNT study). Eur J Cardiovasc Prev Rehabil. 2008 Dec;15(6):639–645. 18779734 10.1097/HJR.0b013e3283101671

[52] Hamer M, Stamatakis E. Physical activity and mortality in men and women with diagnosed cardiovascular disease. Eur J Cardiovasc Prev Rehabil. 2009 Apr;16(2):156–60. doi: 10.1097/HJR.0b013e32831f1b77 19276984

[53] Loprinzi PD, Addoh O. Accelerometer-determined physical activity and all-cause mortality in a national prospective cohort study of adults post-acute stroke. Am J Health Promot. 2018 Jan;32(1):24–27. doi: 10.1177/0890117117720061 28718295

[54] Stewart RAH, Held C, Hadziosmanovic N, et al. Physical activity and mortality in patients with stable coronary heart disease. J Am Coll Cardiol. 2017 Oct 3;70(14):1689–1700. doi: 10.1016/j.jacc.2017.08.017 28958324

[55] Biscaglia S, Campo G, Sorbets E, et al. Relationship between physical activity and long-term outcomes in patients with stable coronary artery disease. Eur J Prev Cardiol. 2020 Mar;27(4):426–436. 31558054 10.1177/2047487319871217

[56] Bakker EA, Lee DC, Hopman MTE, et al. Dose-response association between moderate to vigorous physical activity and incident morbidity and mortality for individuals with a different cardiovascular health status: a cohort study among 142,493 adults from the Netherlands. PLOS Med. 2021 Dec;18(12):e1003845. 34855764 10.1371/journal.pmed.1003845 PMC8638933

[57] Buckley BJR, Lip GYH, Thijssen DHJ. The counterintuitive role of exercise in the prevention and cause of atrial fibrillation. Am J Physiol Heart Circ Physiol. 2020 Nov 1;319(5):H1051–H1058. 32946289 10.1152/ajpheart.00509.2020

[58] Pathak RK, Elliott A, Middeldorp ME, et al. Impact of CARDIOrespiratory FITness on arrhythmia recurrence in obese individuals with atrial fibrillation: the CARDIO-FIT study. J Am Coll Cardiol. 2015 Sep 1;66(9):985–96. doi: 10.1016/j.jacc.2015.06.488 26113406

[59] Proietti M, Boriani G, Laroche C, et al. Self-reported physical activity and major adverse events in patients with atrial fibrillation: a report from the EURObservational Research Programme Pilot survey on atrial fibrillation (EORP-AF) General registry. Europace. 2017 Apr 1;19(4):535–543. doi: 10.1093/europace/euw169 28431068

[60] Garnvik LE, Malmo V, Janszky I, et al. Physical activity, cardiorespiratory fitness, and cardiovascular outcomes in individuals with atrial fibrillation: the HUNT study. Eur Heart J. 2020 Apr 14;41(15):1467–1475. doi: 10.1093/eurheartj/ehaa032 32047884 PMC7320825

[61] Malmo V, Nes BM, Amundsen BH, et al. Aerobic interval training reduces the burden of atrial fibrillation in the short term: a randomized trial. Circulation. 2016 Feb 2;133(5):466–73. doi: 10.1161/CIRCULATIONAHA.115.018220 26733609

[62] O’Connor CM, Whellan DJ, Lee KL, et al. Efficacy and safety of exercise training in patients with chronic heart failure: HF-ACTION randomized controlled trial. JAMA. 2009 Apr 8;301(14):1439–50. doi: 10.1001/jama.2009.454 19351941 PMC2916661

[63] Lawler PR, Filion KB, Eisenberg MJ. Efficacy of exercise-based cardiac rehabilitation post-myocardial infarction: a systematic review and meta-analysis of randomized controlled trials. Am Heart J. 2011 Oct;162(4):571–584 e2. doi: 10.1016/j.ahj.2011.07.017 21982647

[64] Dibben G, Faulkner J, Oldridge N, et al. Exercise-based cardiac rehabilitation for coronary heart disease. Cochrane Database Syst Rev. 2021 Nov 6;11(11):CD001800. doi: 10.1002/14651858.CD001800.pub4 34741536 PMC8571912

[65] Chair SY, Zou H, Cao X. Effects of exercise therapy for adults with coronary heart disease: a Systematic Review and Meta-analysis of Randomized Controlled Trials. J Cardiovasc Nurs. 2021 Jan;36(1):56–77. doi: 10.1097/JCN.0000000000000713 32649373

[66] Andersen K, Farahmand B, Ahlbom A, et al. Risk of arrhythmias in 52 755 long-distance cross-country skiers: a cohort study. Eur Heart J. 2013 Dec;34(47):3624–31. 23756332 10.1093/eurheartj/eht188

[67] La Gerche A, Burns AT, Mooney DJ, et al. Exercise-induced right ventricular dysfunction and structural remodelling in endurance athletes. Eur Heart J. 2012 Apr;33(8):998–1006. 22160404 10.1093/eurheartj/ehr397

[68] Mohlenkamp S, Lehmann N, Breuckmann F, et al. Running: the risk of coronary events: prevalence and prognostic relevance of coronary atherosclerosis in marathon runners. Eur Heart J. 2008 Aug;29(15):1903–10. 18426850 10.1093/eurheartj/ehn163

[69] Myrstad M, Nystad W, Graff-Iversen S, et al. Effect of years of endurance exercise on risk of atrial fibrillation and atrial flutter. Am J Cardiol. 2014 Oct 15;114(8):1229–33. doi: 10.1016/j.amjcard.2014.07.047 25169984

[70] Petek BJ, Churchill TW, Moulson N, et al. Sudden Cardiac Death in National Collegiate Athletic Association Athletes: a 20-year study. Circulation. 2024 Jan 9;149(2):80–90. doi: 10.1161/CIRCULATIONAHA.123.065908 37955565 PMC10843024

[71] Morita H, Asada ST, Miyamoto M, et al. Significance of exercise-related ventricular arrhythmias in patients with Brugada syndrome. J Am Heart Assoc. 2020 Dec;9(23):e016907. 33222599 10.1161/JAHA.120.016907 PMC7763771

[72] Schnohr P, O’Keefe JH, Marott JL, et al. Dose of jogging and long-term mortality: the Copenhagen city heart study. J Am Coll Cardiol. 2015 Feb 10;65(5):411–9. doi: 10.1016/j.jacc.2014.11.023 25660917

[73] Lee DC, Pate RR, Lavie CJ, et al. Leisure-time running reduces all-cause and cardiovascular mortality risk. J Am Coll Cardiol. 2014 Aug 5;64(5):472–81. doi: 10.1016/j.jacc.2014.04.058 25082581 PMC4131752

[74] Armstrong ME, Green J, Reeves GK, et al. Frequent physical activity may not reduce vascular disease risk as much as moderate activity: large prospective study of women in the United Kingdom. Circulation. 2015 Feb 24;131(8):721–729. doi: 10.1161/CIRCULATIONAHA.114.010296 25688148

[75] Heidbuchel H, Hoogsteen J, Fagard R, et al. High prevalence of right ventricular involvement in endurance athletes with ventricular arrhythmias. Role of an electrophysiologic study in risk stratification. Eur Heart J. 2003 Aug;24(16):1473–1480. 12919770 10.1016/s0195-668x(03)00282-3

[76] Batty GD, Shipley MJ, Marmot M, et al. Leisure time physical activity and coronary heart disease mortality in men symptomatic or asymptomatic for ischaemia: evidence from the Whitehall study. J Public Health Med. 2003 Sep;25(3):190–6. 14575192 10.1093/pubmed/fdg043

[77] Mons U, Hahmann H, Brenner H. A reverse J-shaped association of leisure time physical activity with prognosis in patients with stable coronary heart disease: evidence from a large cohort with repeated measurements. Heart. 2014 Jul;100(13):1043–9. doi: 10.1136/heartjnl-2013-305242 24829374

[78] Williams PT, Thompson PD. Increased cardiovascular disease mortality associated with excessive exercise in heart attack survivors. Mayo Clin Proc. 2014 Sep;89(9):1187–94. doi: 10.1016/j.mayocp.2014.05.006 25128072

[79] Tran ZV, Weltman A, Glass GV, et al. The effects of exercise on blood lipids and lipoproteins: a meta-analysis of studies. Med Sci Sports Exerc. 1983;15(5):393–402. doi: 10.1249/00005768-198315050-00009 6645868

[80] Leaf DA. The effect of physical exercise on reverse cholesterol transport. Metabolism. 2003 Aug;52(8):950–7. doi: 10.1016/S0026-0495(03)00147-1 12898457

[81] Wen H, Wang L. Reducing effect of aerobic exercise on blood pressure of essential hypertensive patients: a meta-analysis. Medicine (Baltimore). 2017 Mar;96(11):e6150. doi: 10.1097/MD.0000000000006150 28296729 PMC5369884

[82] Ford ES. Does exercise reduce inflammation? Physical activity and C-reactive protein among U.S. adults. Epidemiology. 2002 Sep;13(5):561–8. doi: 10.1097/00001648-200209000-00012 12192226

[83] Colberg SR, Sigal RJ, Yardley JE, et al. Physical Activity/Exercise and diabetes: a position statement of the American diabetes association. Diabetes Care. 2016 Nov;39(11):2065–2079. 27926890 10.2337/dc16-1728 PMC6908414

[84] Fiuza-Luces C, Santos-Lozano A, Joyner M, et al. Exercise benefits in cardiovascular disease: beyond attenuation of traditional risk factors. Nat Rev Cardiol. 2018 Dec;15(12):731–743. 30115967 10.1038/s41569-018-0065-1

[85] Franklin BA, Thompson PD, Al-Zaiti SS, et al. Exercise-related acute cardiovascular events and potential deleterious adaptations following long-term exercise training: placing the risks into perspective-an update: a scientific statement from the American heart association. Circulation. 2020 Mar 31;141(13):e705–e736. doi: 10.1161/CIR.0000000000000749 32100573

[86] Di Francescomarino S, Sciartilli A, Di Valerio V, et al. The effect of physical exercise on endothelial function. Sports Med. 2009;39(10):797–812. doi: 10.2165/11317750-000000000-00000 19757859

[87] Hambrecht R, Fiehn E, Weigl C, et al. Regular physical exercise corrects endothelial dysfunction and improves exercise capacity in patients with chronic heart failure. Circulation. 1998 Dec 15;98(24):2709–15. doi: 10.1161/01.CIR.98.24.2709 9851957

[88] Aengevaeren VL, Mosterd A, Sharma S, et al. Exercise and coronary atherosclerosis: observations, explanations, relevance, and clinical management. Circulation. 2020 Apr 21;141(16):1338–1350. doi: 10.1161/CIRCULATIONAHA.119.044467 32310695 PMC7176353

[89] Chen H, Chen C, Spanos M, et al. Exercise training maintains cardiovascular health: signaling pathways involved and potential therapeutics. Signal Transduct Target Ther. 2022 Sep 1;7(1):306. doi: 10.1038/s41392-022-01153-1 36050310 PMC9437103

[90] Moreira JBN, Wohlwend M, Wisloff U. Exercise and cardiac health: physiological and molecular insights. Nat Metab. 2020 Sep;2(9):829–839. doi: 10.1038/s42255-020-0262-1 32807982

[91] Mucke M, Ludyga S, Colledge F, et al. Influence of regular physical activity and fitness on stress reactivity as measured with the trier social stress test protocol: a systematic review. Sports Med. 2018 Nov;48(11):2607–2622. 30159718 10.1007/s40279-018-0979-0

[92] Kline CE. The bidirectional relationship between exercise and sleep: implications for exercise adherence and sleep improvement. Am J Lifestyle Med. 2014 Nov;8(6):375–379. doi: 10.1177/1559827614544437 25729341 PMC4341978

[93] Nambiema A, Lisan Q, Perier MC, et al. Healthy sleep score and incident cardiovascular diseases: the Paris prospective study III (PPS3). Eur Heart J. 2022;43(Supplement_2). doi: 10.1093/eurheartj/ehac544.2451 PMC10719494 37860848

[94] Schneider RH, Grim CE, Rainforth MV, et al. Stress reduction in the secondary prevention of cardiovascular disease: randomized, controlled trial of transcendental meditation and health education in blacks. Circ Cardiovasc Qual Outcomes. 2012 Nov;5(6):750–8. 23149426 10.1161/CIRCOUTCOMES.112.967406 PMC7269100

[95] Mikus CR, Boyle LJ, Borengasser SJ, et al. Simvastatin impairs exercise training adaptations. J Am Coll Cardiol. 2013 Aug 20;62(8):709–14. doi: 10.1016/j.jacc.2013.02.074 23583255 PMC3745788

[96] Shave R, Baggish A, George K, et al. Exercise-induced cardiac troponin elevation: evidence, mechanisms, and implications. J Am Coll Cardiol. 2010 Jul 13;56(3):169–76. doi: 10.1016/j.jacc.2010.03.037 20620736

[97] Thompson PD, Franklin BA, Balady GJ, et al. Exercise and acute cardiovascular events placing the risks into perspective: a scientific statement from the American Heart Association Council on Nutrition, physical activity, and metabolism and the Council on clinical cardiology. Circulation. 2007 May 1;115(17):2358–68. doi: 10.1161/CIRCULATIONAHA.107.181485 17468391

[98] Vogel B, Acevedo M, Appelman Y, et al. The lancet women and cardiovascular disease commission: reducing the global burden by 2030. Lancet. 2021 Jun 19;397(10292):2385–2438. doi: 10.1016/S0140-6736(21)00684-X 34010613

[99] Bucciarelli V, Mattioli AV, Sciomer S, et al. The impact of physical activity and inactivity on cardiovascular risk across Women’s lifespan: an updated review. J Clin Med. 2023 Jun 28;12(13):4347. doi: 10.3390/jcm12134347 37445383 PMC10342501

[100] Knuuti J, Wijns W, Saraste A, et al. 2019 ESC guidelines for the diagnosis and management of chronic coronary syndromes. Eur Heart J. 2020 Jan 14;41(3):407–477. doi: 10.1093/eurheartj/ehz425 31504439

[101] Pelliccia A, Sharma S, Gati S, et al. 2020 ESC Guidelines on sports cardiology and exercise in patients with cardiovascular disease. Eur Heart J. 2021 Jan 1;42(1):17–96. doi: 10.1093/eurheartj/ehaa605 33180902

[102] Virani SS, Newby LK, Arnold SV, et al. 2023 AHA/ACC/ACCP/ASPC/NLA/PCNA guideline for the management of patients with chronic coronary disease: a report of the American Heart Association/American College of Cardiology Joint Committee on clinical practice guidelines. Circulation. 2023 Aug 29;148(9):e9–e119. doi: 10.1161/CIR.0000000000001168 37471501

[103] Visseren FLJ, Mach F, Smulders YM, et al. 2021 ESC Guidelines on cardiovascular disease prevention in clinical practice. Eur Heart J. 2021 Sep 7;42(34):3227–3337. doi: 10.1093/eurheartj/ehab484 34458905

[104] Abreu A, Frederix I, Dendale P, et al. Standardization and quality improvement of secondary prevention through cardiovascular rehabilitation programmes in Europe: the avenue towards EAPC accreditation programme: a position statement of the secondary prevention and rehabilitation Section of the European Association of Preventive Cardiology (EAPC). Eur J Prev Cardiol. 2021 May 14;28(5):496–509. doi: 10.1177/2047487320924912 33611459

[105] Ruivo J, Moholdt T, Abreu A. Overview of cardiac rehabilitation following post-acute myocardial infarction in European Society of Cardiology member countries. Eur J Prev Cardiol. 2023 Jul 12;30(9):758–768. 36722203 10.1093/eurjpc/zwad024

